# Employment-related mental health outcomes among Australian migrants: A 19-year longitudinal study

**DOI:** 10.1177/00048674231174809

**Published:** 2023-05-21

**Authors:** Humaira Maheen, Tania King

**Affiliations:** Centre for Health Equity, Melbourne School of Population and Global Health, The University of Melbourne, Carlton, VIC, Australia

**Keywords:** Migrant, employment, mental health, Asian, African, Middle Eastern, Australia

## Abstract

**Background:**

Migrants experience various stressors at different stages of migration based on their country of origin, ethnic backgrounds, migration context and host country. Employment is one important post-settlement factor associated with mental health among migrant groups. The study investigates whether the country of origin modifies the association between employment and mental health for Australian migrants.

**Methods:**

Nineteen waves of data from the Household Income and Labour Dynamics in Australia Survey were used. Using fixed-effects regression, we examined the effects of within-person changes in employment status on mental health outcomes as measured by the Mental Health Inventory (MHI-5), controlling for time-varying confounders and stratified by sex and examined effect modification by country of origin.

**Results:**

The relationship between unemployment and mental health was modified by country of origin for men but not women. Unemployed men from Asian (β = −4.85, *p* < 0.001), African and Middle Eastern (β = −3.61, *p* < 0.05) countries had lower mental health scores compared to employed Australian-born men. For men, there was evidence of effect modification of the association between employment and mental health by country of origin, with the combined effect of being unemployed and being a migrant from an Asian country was almost three points lower than the summed independent risks of these factors (β = −2.72; *p* = 0.01). Also, for men, the combined mental health effect of not being in the labour force and coming from a non-English-speaking European country was greater than the summed effects of these factors (β = −2.33; *p* < 0.001).

**Conclusion:**

Tailored employment-support programmes may be beneficial for migrants from ethnic minorities, particularly those from Asian, African and Middle Eastern countries in Australia. Further research is needed to understand why the mental health of migrant men from these countries is particularly vulnerable to unemployment.

## Introduction

The past century has seen a significant increase in the number of people migrating internationally. According to the World Migration Report 2022, there are approximately 272 million international migrants worldwide, which is 3.5% of the world’s population (International Organization for Migration, 2022). Migration is now recognised as a social determinant of health (Davies et al., 2009; Ingleby, 2012) as not only the factors that drive migration can cause health inequalities; some migrants may experience social disadvantages in their host countries that can increase the risk of poor health. With the growing migration population around the world, it is crucial to understand the unique health needs and risk factors of different migrant groups in order to support global population health.

The relationship between migration and mental health is well-established. Studies have shown that migrants from Asia, Africa and Middle Eastern countries who have settled in Western countries experience poorer mental health compared to the non-migrant population in their host countries (Close et al., 2016; Jurado et al., 2017). However, there are subtle differences based on their country of origin, reason of migration and the socio-demographic profile of individual migrants (Jurado et al., 2017). For instance, refugee migrants from these countries show a high prevalence of diagnosed and self-reported anxiety (13–42%), depression (30–40%) and post-traumatic stress disorder (PTSD) (29–37%) in their host countries, compared to non-refugee population globally, as well as non-refugee population living in conflict and war setting (Henkelmann et al., 2020). Similar findings have been reported for African migrants (James et al., 2022) and Syrian refugees (Nguyen et al., 2022) living in Western countries compared to their host population. These disparities can be attributed in part to their previous exposure to conflict or war, limited income means at the time of migration or settlement-related stressors such as securing housing or employment (Chen et al., 2017; Steel et al., 2002). Economic migrants, while having some advantages over refugees, such as voluntary migration and the ability to return to their home countries, may face other settlement challenges, such as difficulty finding relevant work (Axelsson, 2017), cultural adaptation difficulties (Mwanri et al., 2022) and discrimination based on their ethnic or cultural backgrounds (Abdelkerim and Grace, 2012; Maneze et al., 2014). There is some evidence that acculturation stress may also contribute to poor mental health for migrants from ethnically diverse backgrounds (Milner and Khawaja, 2010; Oh et al., 2002; Thomas, 1995). For those, who migrate from collectivist to individualistic societies, acculturation stress can result in an actual or perceived lack of adequate social support, a disparity between expectations and reality and low self-esteem (Bhugra and Becker, 2005) which then can lead to poor mental health in both economic and refugee migrants.

Employment is a crucial factor in the settlement process for migrant populations. Ethnic minorities in Western societies tend to be disproportionately represented in low-paying and insecure jobs, which can lead to increased rates of work-related injuries and sickness presenteeism which can have a significant impact on both physical and mental health (Moyce and Schenker, 2018; Sterud et al., 2018). In Australia, ethnic minorities are also under-represented in high-level positions of authority (Soutphommasane et al., 2018) and are at a higher risk of experiencing psychosocial stressors in low-skilled jobs (Daly et al., 2018). Migrants from Asian (Daly et al., 2018; Maneze et al., 2014; Steel et al., 2002), African (Abdelkerim and Grace, 2012; Mwanri et al., 2022) and Middle Eastern countries (Chen et al., 2017; Daly et al., 2018) or refugee migrants from these countries (Wu et al., 2021) face various barriers to obtaining employment, and these barriers may vary based on factors such as gender, age, language proficiency and length of stay. These employment-related stressors can have a significant impact on the mental health of these migrants.

Australia is a culturally diverse country that accepts a considerable number (per capita) of international migrants every year. According to the 2021 Australian Census, one in four Australians were born overseas, and half have a parent born overseas. Despite this diversity, research on the relationship between employment and mental health among migrants in Australia is limited. Most studies are based on qualitative data (Mwanri et al., 2022), cross-sectional data (Daly et al., 2018; Straiton et al., 2014) or are restricted to specific groups of migrants, such as refugees (Chen et al., 2017; Steel et al., 2002; Wu et al., 2021). To fill this research gap, we used 19-year national-level longitudinal data to examine the relationship between employment and mental health in migrant groups. The study aims to investigate whether migrants’ country of origin modifies the association between employment and mental health among both men and women in the Australian population.

## Methods

### Data source

The Household, Income and Labour Dynamics in Australia (HILDA) Survey is a nationally representative longitudinal study of Australian households, which collects information about economic and personal well-being, labour market dynamics and family life since 2001. We used data from individuals aged 15–64 years to represent the working-age population. The response rates for the survey are above 80%, with a 6% attrition rate. Detailed information about the data collection methods, sampling techniques and survey items has been comprehensively described elsewhere (Melbourne Institute Applied Economics and Research, 2022).

### Exposure variable – employment

Employment status was assessed as a categorical variable: employed, unemployed (actively looking for work) and not in the labour force (NILF). The NILF category included people who are not actively looking for work, informal carers (of children, elderly parents, or someone with a disability), students, those with health conditions, or retirees.

### Outcome variable – mental health

The Mental Health Inventory (MHI-5) was used to assess the population’s mental health. The MHI-5 is a 5-item subscale from the Short Form-36 (SF-36) general health measure, which is annually collected as part of the HILDA’s self-reported questionnaire (SCQ). The MHI-5 assesses symptoms of depression and anxiety (nervousness, depressed affect) and positive aspects of mental health (feeling calm and happy) in the past 4 weeks. It is a validated instrument for screening mood disorders and depressive symptomatology in the general population and has been validated as a measure for depression using clinical interviews as the gold standard (Rumpf et al., 2001). The current analysis used the continuous MHI-5 score (unstandardised scale of 1–100), with higher scores representing better mental health. Previous studies from the HILDA survey have also used unstandardised MHI-5 scores to examine individuals’ mental health over time (Aitken et al., 2020; Milner et al., 2016; Milner and LaMontagne, 2017). Despite the lack of a universal interpretation of MHI-5 scores for clinical significance, a difference of three points on the unstandardised scale is considered clinically significant (Ware, 1993). In this study, we used the definitions of small, moderate and large effects for the MHI-5 score provided by Contopoulos-Ioannidis et al. (2009), who classified differences in mean unstandardised scores as small (less than 4 MHI-5 points), moderate (4–10 points) or large (more than 10 points).

### Effect modifiers – country of origin

We used the country of origin as an effect modifier for our analysis. We classified countries using the Standard Australian Classification of Countries (SACC) Level 1 (Australian Bureau of Statistics, 2016). We grouped migrants from English-speaking countries (ESC; such as New Zealand, England, South Africa, Canada and the United States) into one category. Additionally, due to a low number of participants from Sub-Saharan African countries, we merged them into the North African and Middle East (SACC 4) category. We also combined Oceania and the Americas (excluding North America) for the same reason. Asian and European sub-regions were also combined as separate groups. The revised country of origin categories includes Australia, ESC, Europe (excluding ESC), Asia, Middle East and Africa (excluding South Africa) and Oceania and America (excluding ESC). Table 1 compares the SACC category 1 with the effect modifier used in our analysis.

**Table 1.** Categorisation of countries of origin.

Standard Australian Classification of Countries (SACC) category	Countries of origin used in the present analysis	Notes
1. Oceania and Antarctica	1. Australia	
2. North-West Europe	2. English-speaking countries (ESC)	The United Kingdom, Ireland, the United States, New Zealand, South Africa and Canada
3. Southern and Eastern Europe	3. European countries	SACC 2 and 3, excluding ESC
4. North Africa and the Middle East	4. Asia	SACC 5–7
5. South East Asia	5. Middle East and African countries	SACC 4, 9 (excluding South Africa)
6. North East Asia	6. Oceania and Americas	SACC 1, 8 (excluding North America)
7. Southern and Central Asia		
8. Americas		
9. Sub-Saharan Africa		

### Confounders

**Figure 1.**
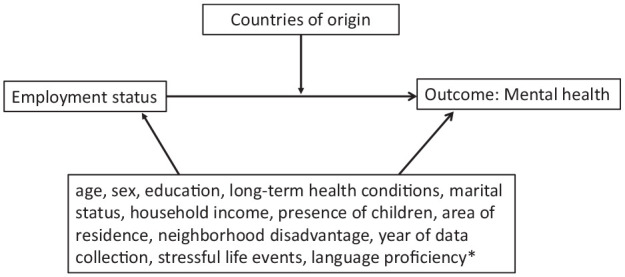
Directed acyclical graph. *Included in sensitivity analysis only.

Based on the literature, the following variables were considered as potential confounders of the relationship between employment and mental health (Milner et al., 2016; Milner and LaMontagne, 2017; Pernice et al., 2009; Wu et al., 2021). The confounders that were used in the models were age (coded continuously), year of data collection (waves), education level, weekly household income (adjusted for household size), presence of long-term health condition, presence of children, place of residence, marital status, length of time spent in Australia, language proficiency, number of stressful life events in the past year and neighbourhood disadvantage as measured by the Socio-Economic Indexes for Areas (SEIFA) Index. Specifically, we used the SEIFA 2011 Decile of the Index of Relative Socio-economic Disadvantage (IRSD), with quintile 1 representing the most disadvantaged neighbourhoods and quintile 5 representing the least disadvantaged (Australian Bureau of Statistics, 2022). From the list of stressful life events, we included serious personal injury/illness, serious injury/illness of a family member, death of a spouse or child or death of a close relative/family member (Milner et al., 2017). Figure 1 shows the directed acyclic graph (DAG) depicting the exposure, outcome and confounders included in our analysis.

### Analytical sample

Our analytical sample included 200,327 observations from 26,095 people aged 15–64 years. Data from the first wave were excluded due to the missing information on stressful life events and language proficiency. Information on language proficiency was also missing in approximately 50% of observations from individuals of non-English-speaking countries throughout waves 2–19, and thus was not included in the main analysis. All other confounding factors had less than 4% missing data.

**Table 2.** Characteristics of analytical sample (observations 200,327, persons 26,095).

Variables	Baseline (wave 2)		Last wave (wave 19)	
	Men (*n* = 4825)	Women (*n* = 5334)	Men (*n* = 5953)	Women (*n* = 6683)
MHI-5^a^ score (Mean, SD)^b^	75.0, 16.7	72.4, 17.6	72.8, 17.6	69.6, 18.7
Employment (%)				
Employed	79.1	65.2	80.2	72.7
Unemployed	4.8	4.1	5.4	4.2
Not in the labour force	16.1	30.8	14.4	23.0
Countries of origin (%)				
Australia	77.8	78.4	82.4	82.3
English-speaking countries	10.6	9.3	7.9	6.6
European countries^c^	4.4	4.6	1.9	2.1
Asia	4.5	5.4	5.0	6.5
Middle East and Africa	1.5	1.2	1.3	1.0
Oceania and Americas^c^	1.3	1.1	1.6	1.4
Age, years (Mean, SD)	38.7, 13.6	38.5, 35.3	38.7, 14.2	39.3, 14.1
Average equivalised annual income	31,945.6	30,484.9	62,701.6	60,513.5
Education (%)				
Postgraduate	7.8	7.7	11.0	14.8
Bachelor	12.0	13.7	14.5	19.4
Diploma or certificate	34.3	20.0	36.9	30.6
Year 12	14.2	16.9	17.3	16.8
Less than year 12	31.7	41.8	20.3	18.5
Place of residence (%)				
Urban	61.5	62.1	62.9	62.8
Rural	38.5	37.9	37.1	37.2
Long-term health condition (%)				
No	80.9	83.7	78.7	76.2
Yes	19.1	16.3	21.3	23.8
Presence of children (%)				
Couple no children	23.7	24.5	24.4	25.3
Couple with children	50.9	48.4	49.7	45.4
Parent with children	6.7	12.3	6.9	12.7
Lone person	13.5	10.5	13.9	11.4
Other	5.2	4.3	5.2	5.2
Stressful life event (injury/illness)				
No	78.6	76.0	82.5	79.4
Yes	21.4	24.0	17.5	20.6
Stressful life event (death)				
No	80.3	79.7	82.9	81.8
Yes	19.7	20.3	17.1	18.2
Socio-Economic Indexes for Areas (SEIFA)				
Quintile 1	19.7	20.0	17.8	17.7
Quintile 2	20.6	20.1	19.1	19.2
Quintile 3	19.6	19.4	20.2	20.5
Quintile 4	19.0	19.2	21.6	21.8
Quintile 5	21.1	21.3	21.3	20.8

a MHI-5: Mental Health Inventory.

b SD: standard deviation.

c Excluding English-speaking countries.

### Sensitivity analysis

We performed a sensitivity analysis by classifying the country of origin according to the SACC Level 1, excluding the Middle East from North Africa (Table 2.1 in Supplementary File 2). Additionally, considering the theoretical significance of language proficiency, we conducted a sensitivity analysis including language proficiency as a confounder (Table 2.2 in Supplementary File 2).

### Statistical analysis

Descriptive statistics were calculated to examine the characteristics of the analytical sample for men and women for the baseline (wave 2) and the last wave (wave 19). Longitudinal linear regression models with fixed-effect estimators were used to study the association between employment and mental health, specifying respondents (respondent ID) as the panel variable and year as the time variable. Fixed-effects linear regression models analyse the within-person change in outcome in relation to change in exposure, with each person acting as their own control (Gunasekara et al., 2014). Fixed-effects models are particularly useful to control for the bias caused by time-invariant confounders (e.g. personality factors) in causal estimates. These models remove person-stable biases and therefore provide a more robust test of the association between exposure and the outcome. In analyses such as these, where pre-migration experiences are unmeasured but are likely to vary considerably across individuals, fixed-effects approaches are ideal as they mitigate any bias associated with such unmeasured characteristics. It is also important to note that fixed-effects models typically represent data in a way that is more realistic as they are substantially less restrictive than random effects models (Allison, 2014). In this study, fixed-effects models measured average within-person changes in a person’s mental health in relation to changes in employment status, with employed as the reference category.

We then tested the association for effect modification by country of origin. The model with and without the interaction was compared using the likelihood ratio test to assess evidence of effect modification. The post-estimation command from Stata 16.0 (lincom) was used to estimate linear combinations of regression coefficients for significant interactions. All statistical analyses were performed using Stata 16 statistical package [50]. Two separate regression models (without and with interactions) were fitted for both men and women.

## Results

The characteristics of the analytical sample for men and women are presented in Table 2, including information from the baseline (wave 2) and the final wave (wave 19). The MHI-5 scores showed a decline for both men and women over time. At baseline, 80% of men in the sample were employed, while the percentage of employed women increased from 65% to 73% by the final wave. When comparing the proportion of respondents participating in waves 2 and 19 based on country of origin, there was a slight increase in the number of respondents from Asian, African and Middle Eastern countries as well as those born in Australia. This is likely due to the addition of a top-up sample in 2011. The most notable decrease in observations was seen in the migrant category of European countries, which dropped from 4.4% to 1.9%. This could be due to the sample being limited to individuals between the ages of 15 and 64 years and the presence of more mature European migrants in earlier waves. Overall, the sample became more educated over time, with a 2% increase in long-term health conditions from wave 2 to wave 19.

Table 3 shows the average MHI-5 score by country of origin for men and women. For all countries of origin, employed individuals have higher average MHI-5 scores compared to unemployed and those NILF, for both men and women. Overall, the average scores did not vary substantially across different countries of origin in each employment category.

**Table 3.** Average mental health inventory (MHI)-5 score by countries of origin for men and women.

Mental Health Inventory (MHI) score	Employed (M, SD)	Unemployed (M, SD)	NILF (M, SD)	Employed (M, SD)	Unemployed (M, SD)	NILF (M, SD)
	Men			Women		
Australia	76.2, 15.5	67.8, 19.3	68.4, 20.8	73.6, 16.3	64.3, 20.3	68.3, 20.6
English-speaking countries	76.0, 15.6	69.6, 17.7	69.9, 20.7	75.4, 15.9	69.8, 20.5	69.5, 20.0
European countries^a^	75.4, 15.2	67.7, 17.6	67.3, 20.6	72.8, 17.2	65.1, 19.2	66.8, 18.4
Asia	74.5, 16.2	68.4, 18.5	69.6, 17.6	73.2, 15.3	66.9, 18.6	71.5, 17.9
Middle East and Africa	76.2, 16.0	71.5, 17.1	60.7, 22.4	72.7, 16.7	67.3, 19.2	65.7, 20.2
Oceania and Americas^a^	74.8, 16.3	67.6, 18.5	66.3, 19.3	73.7, 17.9	65.1, 21.4	67.0, 19.1

SD: standard deviation; NILF: not in the labour force.

a Excluding English-speaking countries.

The results of our fixed-effects regression analysis, including both main and interaction models for men, are presented in Supplementary File 1. Our analysis showed a significant interaction between ethnicity and employment for men, as determined by the likelihood ratio test (χ^2^ = 24.3, *p* < 0.05), but not for women (χ^2^ = 5.92, *p* = 0.82). The association between mental health and employment was not found to be significantly different for migrant men from ESC compared to Australian-born.

Table 4 presents the additive effect measure modification of unemployment and NILF on mental health by country of origin for men from non-English-speaking countries. The stratum-specific results for unemployment showed that migrant men from Asian countries reported mental health scores an average of five points lower than employed Australian-born men, whereas those from African and Middle Eastern countries reported an almost four-point lower mental health score when unemployed compared to employed Australian-born men. Based on the Contopoulos-Ioannidis et al. (2009) recommendation on unstandardised scores, we determined that the stratum-specific results for unemployment demonstrated at least a moderate effect on the mental health of migrant men from Asian, African and Middle Eastern countries. There was evidence of effect modification by country of origin: for men, the combined effect of being unemployed and coming from an Asian country on mental health was almost three points more than the summed independent effects of unemployment and Asian country of origin (β = −2.72; 95% confidence interval [CI] = [–4.80, −0.63]; *p* = 0.01).

**Table 4.** Effect modification of the relationship between employment and mental health by countries of origin for non-English-speaking countries for men.

Mental Health Inventory (MHI) score	Australia	Asia	Africa and Middle East (excluding South Africa)	Europe excluding English-speaking countries	America and Oceania (excluding North America)
Unemployment					
Employed	Reference	–	–	–	–
Unemployed	−2.13 [−2.59, 1.67]***	−4.85 [−6.89, −2.81]***	−3.61 [−6.84, −0.38]*	−2.88 [−6.02, 0.26]	0.83 [−2.33, 4.00]
EMM		−2.72[−4.80, −0.63]*	−1.48 [−4.74, 1.79]	−0.75[−3.93, 2.42]	2.96 [−0.24, 6.16]
NILF status					
Employed	Reference	–	–	–	
NILF	−1.53 [−1.88, −1.17]***	−0.79 [−2.38, −0.81]	−2.17 [−5.02, 0.68]	−3.86 [−5.92, −1.80]***	−2.21 [−4.81, 0.38]
EMM		0.74 [−0.89, 2.37]	−0.64 [−3.52, 2.23]	−2.33 [−4.42, −0.24]*	−0.69 [−3.30, 1.93]

EMM: effect measure modification; NILF: not in the labour force.

* *p* < 0.05; ***p < 0001.

There was a moderate effect of NILF status on the mental health of European migrant men, whose mental health score was four points lower compared to employed Australian-born men. Effect modification was also observed for European migrants with NILF status: the combined effect of NILF and being a migrant from a non-English-speaking European country was more than two points lower (β = −2.33; 95% CI = [−4.42, −0.24]; *p* < 0.001) than the combined risks of being NILF and from a non-English-speaking European country.

The HILDA survey collected data on the reasons for NILF for the past 4 weeks. The results, presented in Supplementary File 3, show the five most common reasons reported by men. About 32% of European men reported being NILF due to their own health conditions, compared to 19% of men from Asian countries, 37% from African and Middle Eastern countries, 45% from Oceania and the Americas and 32% were Australian-born. The most common reason reported by Asian (42%) and Australian-born men (37%) for being NILF was studying or returning to studies.

### Sensitivity analysis

We conducted a sensitivity analysis by including language proficiency as a confounding variable in the model, using a sample of 190,409 observations (Supplementary File 2). The results indicate a larger effect size (similar results) when language was included as a confounder. This was similarly observed using detailed country of origin classification, noting that men from South East Asia and Middle Eastern countries may experience a greater decline in mental health during unemployment compared to unemployed Australian men.

## Discussion

This study presents the first longitudinal evidence of how the relationship between employment and mental health in Australia can vary according to country of origin among both men and women. It shows that men from Asian, African and Middle Eastern countries reported poorer mental health outcomes when unemployed, whereas men from non-English-speaking European countries showed poorer mental health outcomes when NILF. For men, we find evidence that the association between unemployment and mental health is modified by country of origin, with the combined effect of being unemployed and coming from an Asian country being greater than the summed independent effects. For women, there was no evidence of effect modification of the association of mental health and employment by country of origin.

### Unemployment and migrant mental health

Previous studies noted poor mental health of unemployed migrants living in Western countries. This includes Turkish migrants in the Netherlands (Bengi-Arslan et al., 2002), Southeast Asians in Canada (Beiser and Hou, 2001; Beiser and Hou, 2006), Somali refugees in London (Warfa et al., 2012), South Asians in Canada (Dean and Wilson, 2009), Vietnamese migrants in Germany (Wolf et al., 2020), Asian Americans (John et al., 2012), Kurdish migrants in Finland (Rask et al., 2016), African refugees in the United States (Abdelkerim and Grace, 2012) and Indian and Chinese migrants in New Zealand (Pernice et al., 2009). Previous evidence has highlighted how unemployment affects the mental health of immigrant men, especially those from ethnic minorities, and has revealed that these effects differ from those observed in host populations. Unemployment affects male migrants’ identity as breadwinners and full-time workers, and losing that status can poorly affect their mental health (Dean and Wilson, 2009). There may also be additional pressures for these workers, as highlighted by the work of Beiser et al. (1993), who showed that some Southeast Asian refugee men supported their overseas families in addition to their direct families in Canada. Losing their jobs, therefore, can be more stressful for these men as a sense of double incompetence in their inability to provide for both families. This group was also found to experience poor mental health in response to unemployment in this study. This is likely to be different to the experiences of non-immigrant men, who, as part of a more individualistic society, do not carry the financial burden of extended families.

Studies from Australia (Abdelkerim and Grace, 2012), Canada (Beiser and Hou, 2001; Dean and Wilson, 2009) and Sweden (Hollander, 2013) have also shown that some immigrant groups, especially those from Asian, African and Middle Eastern countries, experience additional challenges in obtaining employment. These challenges can range from lack of recognition of their qualifications and previous work experience, communication challenges related to lower language proficiency and having a foreign accent, racial minority background and visa/legal status in their host countries (Abdelkerim and Grace, 2012; Warfa et al., 2012). Research on employment challenges faced by African communities in Australia has highlighted that these stressors, combined with a lack of culturally specific employment services, make the job-seeking process particularly difficult for African migrants (Abdelkerim and Grace, 2012). A recent study from four Organization for Economic Co-operation and Development (OECD) countries has shown racial discrimination by employers against non-European migrants, with the highest racial discrimination rates found in France (85%), Sweden (65%), the United Kingdom (55%) and Canada (44%) (Quillian et al., 2019). These challenges make employment more difficult and can result in unemployment, accepting employment below their skill levels or working in precarious conditions. These experiences can have profound effects on migrants’ mental health. Skilled immigrants from Canada with unemployment or underemployment experiences reported regretting their decision to migrate to a country where their skill set and qualifications are not recognised (Dean and Wilson, 2009). The psychological impact of unemployment can be intensified among men with families overseas or those with limited social networks (Beiser et al., 1993; Beiser and Hou, 2006). Given the employment difficulties that immigrants from Asian, African and Middle Eastern countries often experience in Western countries, including Australia, when they are unemployed, they may fear long-term joblessness and an inability to find relevant work, both of which can negatively impact their mental health, which may explain our findings. Moreover, for many immigrants, work is the way they form social connections and interact with others; losing that connection, in addition to loss of social status and income, can negative affect their mental health.

Depression is one of the most common symptoms experienced by unemployed Asian men (Beiser et al., 1993; Bengi-Arslan et al., 2002; Disney, 2021; John et al., 2012; Warfa et al., 2012; Wolf et al., 2020). In some Western countries, unemployed migrant men from ethnic minority countries are more likely to report generalised anxiety disorder (GAD) (Disney, 2021), hopelessness, social dysfunction, somatic symptoms (Bengi-Arslan et al., 2002), PTSD (Disney, 2021) in response to lengthy job searches and inability to find employment in their relevant skill set (Axelsson, 2017; Dean and Wilson, 2009). What is more problematic is that research shows a lack of mental health service use by migrant men in most Western countries. Reasons associated with poor service use can range from a limited understanding of mental health services in host countries, cost of care, stigma attached to mental health or help-seeking or lack of knowledge of their own mental health (Roberts et al., 2018). An Australian study (Logan et al., 2017) demonstrated a lack of uptake of mental health services for migrant men from non-English-speaking countries according to their need. The combination of poor mental health and a lack of service use can cause long-term negative consequences for the mental health of men from Asian, African and Middle Eastern countries.

Findings from this study highlight the need for effective strategies to address employment-related mental health inequalities for migrant men in Australia. There is a need to evaluate current Australian models of employment support for the migrant population and how these can be optimised to support migrant men during unemployment. It is important to note that most Australian settlement and migrant organisations offer job-seeking support to newly arrived migrants; there is little to no support from migrant organisations to long-term migrants. While the early years of settlement support are arguably the most important, offering ongoing job-seeking support for Asian, African and Middle Eastern migrants may be beneficial. The support may range from upscaling existing programmes to offering new initiatives that have been positively evaluated and shown to be useful elsewhere. For instance, some migrant organisations pair professional mentors with newly arrived migrants to guide them through the employment journey (AMES Australia, 2018; Community Refugee Sponsorship Australia, 2021). Evaluation of these programmes found satisfactory experiences for both mentees and mentors as it helped mentees build confidence, network and understand the dynamics of the Australian labour market and was simultaneously rewarding for mentors (AMES Australia, 2018; Community Refugee Sponsorship Australia, 2021). Offering subsidised job-training programmes and courses for unemployed people in their relevant fields is also an effective way to improve job skills and knowledge. Currently, some programmes in Australia (Department of Education, 2022) are offered for people looking for work. However, their feasibility in helping individuals’ career goals and employment trajectories for migrants, especially those from Asian, African and Middle Eastern countries, needs to be evaluated.

### NILF status and migrant mental health

Our research indicates that while Asian, African and Middle Eastern men’s mental health does not deteriorate when they are not in employment (NILF), European migrant men’s mental health does deteriorate in this circumstance. This is likely due to different reasons for NILF among these groups, with Asian men primarily being in the NILF category for ‘studying’ reasons. In contrast, other factors such as disability from long-term injuries, caring-related fatigue, income loss and dissatisfaction with being away from work may explain poor mental health among other migrant groups. Additionally, the European migrant group had a higher proportion of older individuals, which may contribute to the higher rate of age-related disability in this group.

### Strengths and limitations

The study is the first to employ longitudinal data to examine migrant mental health in relation to employment. Previous Australian studies that used the HILDA data set have commonly used blunter migrant categories (English-speaking and non-English-speaking background), which may not capture the experiences of migrant groups with diverse backgrounds.

In terms of limitations, we acknowledge that the data set may under-represent some migrant groups within the Australian population. Furthermore, while the analysis groups used in this study were based on the SACC Level 1 (Australian Bureau of Statistics, 2016), they may not accommodate the heterogeneity within these groups in terms of cultural and language diversities. Visa categories, which provide a useful proxy measure of migration, were also missing or not asked from many respondents, and were therefore not included in the analysis.

We recognise that an Australian-born group is diverse, and people from different countries (i.e. migrant descendants) are represented in this group. We argue that they may not necessarily experience migration-related challenges (language proficiency, qualification recognition) in relation to job-seeking as those who were born overseas. Similarly, their experiences of racism or discrimination may be different from those born overseas. Since this study focused on employment-related inequalities related to migration, the analysis was restricted to overseas-born migrants. Furthermore, due to the small number of individual country data, we lacked the power to investigate differences by individual country of birth.

## Conclusion

This study highlights the negative impact of unemployment on the mental health of migrant men from Asian, African and Middle Eastern countries, with evidence that the intersection between unemployment and country of origin is especially damaging for the mental health of men from Asian countries. Further research is needed to understand why the mental health of migrant men from these countries is particularly vulnerable to unemployment. There is a need to evaluate current Australian employment-support models for the migrant population and how these can be optimised to support both recently arrived and long-term migrant men during unemployment.

## Supplemental Material

sj-docx-1-anp-10.1177_00048674231174809 – Supplemental material for Employment-related mental health outcomes among Australian migrants: A 19-year longitudinal study Click here for additional data file. Supplemental material, sj-docx-1-anp-10.1177_00048674231174809 for Employment-related mental health outcomes among Australian migrants: A 19-year longitudinal study by Humaira Maheen and Tania King in Australian & New Zealand Journal of Psychiatry

sj-docx-2-anp-10.1177_00048674231174809 – Supplemental material for Employment-related mental health outcomes among Australian migrants: A 19-year longitudinal study Click here for additional data file. Supplemental material, sj-docx-2-anp-10.1177_00048674231174809 for Employment-related mental health outcomes among Australian migrants: A 19-year longitudinal study by Humaira Maheen and Tania King in Australian & New Zealand Journal of Psychiatry

sj-docx-3-anp-10.1177_00048674231174809 – Supplemental material for Employment-related mental health outcomes among Australian migrants: A 19-year longitudinal study Click here for additional data file. Supplemental material, sj-docx-3-anp-10.1177_00048674231174809 for Employment-related mental health outcomes among Australian migrants: A 19-year longitudinal study by Humaira Maheen and Tania King in Australian & New Zealand Journal of Psychiatry
