# Supplementary material for: Employment-related mental health outcomes among Australian migrants: A 19-year longitudinal study
Source: Aust N Z J Psychiatry. 2023 May 21;57(11):1475–85. doi: 10.1177/00048674231174809 (PMC10619185; doi:10.1177/00048674231174809)
Supplement: sj-docx-1-anp-10.1177_00048674231174809 – Supplemental material for Employment-related mental health outcomes among Australian migrants: A 19-year longitudinal study [file sj-docx-1-anp-10.1177_00048674231174809.docx]

**Supplementary file 1**

**Table 1.1 Main Effect Model and Interaction Models for Employment and Mental Health by Countries of Origin using a Fixed-Effects Regression Model (For men)**

| Variables | Main effect | | | Interaction | | |
| --- | --- | --- | --- | --- | --- | --- |
|  | Coefficient | 95% CI | P value | Coefficient | 95% CI | P-value |
| **Employment Status** |  |  |  |  |  |  |
| Employed | Ref |  |  | Ref |  |  |
| Unemployed | -2.17 | -2.59, -1.75 | <0.001 | -2.13 | -2.59, -1.67 | <0.001 |
| NILF | -1.53 | -1.85, -1.20 | <0.001 | -1.53 | -1.88, -1.17 | <0.001 |
|  |  |  |  |  |  |  |
| **Migrant background x employment status^b^** |  |  |  |  |  |  |
| Employed Australian-born |  |  |  | Ref |  |  |
| Unemployed# English-speaking countries |  |  |  | 0.88 | -0.74, 2.50 | 0.29 |
| Unemployed#European countries excluding ESC |  |  |  | -0.75 | -3.93, 2.42 | 0.64 |
| Unemployed#Asia |  |  |  | -2.72 | -4.80, -0.63 | 0.01 |
| Unemployed#Middle East and Africa |  |  |  | -1.48 | -4.74, 1.79 | 0.38 |
| Unemployed#Oceania and Americas excluding ESC |  |  |  | 2.96 | -0.24, 6.16 | 0.07 |
| NILF#English speaking countries |  |  |  | 0.57 | -0.60, 1.74 | 0.34 |
| NILF#European countries excluding ESC |  |  |  | -2.33 | -4.42, -0.24 | 0.03 |
| NILF#Asia |  |  |  | 0.74 | -0.89, 2.37 | 0.37 |
| NILF#Middle East and Africa |  |  |  | -0.64 | -3.52, 2.23 | 0.66 |
| NILF#Oceania and Americas excluding ESC |  |  |  | -0.69 | -3.30, 1.93 | 0.61 |

a The outcome was the MHI-5 score. The models evaluated data from **13,031** persons and **94,173** observations; on average, participants contributed to 7.2 waves of data collection. The models controlled for age, education, marital status, long-term health conditions, household structure, place of residence, year of data collection, injury (self/family), death (spouse/family), and neighbourhood disadvantage. Fixed characteristics such as migrant background were dropped from the main model because that did not change over time.

b Upper and lower confidence intervals at 95% significance.

chi2(6) = 24.3, p-value = 0.0068

**Table 1.2 Main Effect Model for Employment and Mental Health using a Fixed-Effects Regression Model (For women)**

| Variables | Main effect | | | | |
| --- | --- | --- | --- | --- | --- |
|  | Coefficient | 95% CI | | P value | |
| **Employment Status** |  |  | |  | |
| Employed | Ref |  | |  | |
| Unemployed | -1.11 | -1.55, -0.66 | | <0.001 | |
| NILF | -0.69 | -0.94, -0.44 | | <0.001 | |
|  |  | |  | |  |

a The outcome was the MHI-5 score. The models evaluated data from **13,786** persons and **106,154** observations; on average, participants contributed to 7.7 waves of data collection. The models controlled for age, education, marital status, long-term health conditions, household structure, place of residence, year of data collection, injury (self/family), death (spouse/family), and neighbourhood disadvantage. Fixed characteristics such as migrant background were dropped from the main model because that did not change over time.

b Upper and lower confidence intervals at 95% significance.

c Referent for the interaction: employed and Australian-born

Evidence of effect modification was not found.

chi2(6) = 5.92, p-value = 0.82
