# Supplementary material for: Employment-related mental health outcomes among Australian migrants: A 19-year longitudinal study
Source: Aust N Z J Psychiatry. 2023 May 21;57(11):1475–85. doi: 10.1177/00048674231174809 (PMC10619185; doi:10.1177/00048674231174809)
Supplement: sj-docx-2-anp-10.1177_00048674231174809 – Supplemental material for Employment-related mental health outcomes among Australian migrants: A 19-year longitudinal study [file sj-docx-2-anp-10.1177_00048674231174809.docx]

**Supplementary file_2: Sensitivity analyses**

**Table 2.1 Main Effect Model and Interaction Models for Employment and Mental Health by Countries of Origin (For men, including language as a confounder)**

| Variables | Main effect | | | Interaction | | |
| --- | --- | --- | --- | --- | --- | --- |
|  | Coefficient | 95% CI | P value | Coefficient | 95% CI | P-value |
| **Employment Status** |  |  |  |  |  |  |
| Employed | Ref |  |  | Ref |  |  |
| Unemployed | -2.15 | -2.58, -1.72 | <0.001 | -2.13 | -2.59 -1.67 | <0.001 |
| NILF | -1.54 | -1.87,-1.21 | <0.001 | -1.52 | -1.88 -1.17 | <0.001 |
| **Migrant background x employment status** |  |  |  |  |  |  |
| Employed ESC |  |  |  | Ref |  |  |
| Uemployed# English-speaking countries |  |  |  | 0.89 | -0.73, 2.51 | 0.28 |
| Uemployed#European countries excluding ESC |  |  |  | 0.72 | -3.43, 4.87 | 0.73 |
| Umployed#Asia |  |  |  | -2.65 | -5.12, -0.18 | 0.04 |
| Uemployed#Middle East and Africa |  |  |  | -3.94 | -8.02, 0.14 | 0.06 |
| Uemployed#Oceania and Americas excluding ESC |  |  |  | 3.73 | -0.66, 8.11 | 0.10 |
| NILF# English-speaking countries |  |  |  | 0.58 | -0.59, 1.76 | 0.33 |
| NILF#European countries excluding ESC |  |  |  | -4.70 | -7.61, -1.78 | 0.00 |
| NILF#Asia |  |  |  | 0.89 | -0.95, 2.73 | 0.34 |
| NILF#Middle East and Africa |  |  |  | -2.66 | -6.42, 1.11 | 0.17 |
| NILF#Oceania and Americas excluding ESC |  |  |  | -1.78 | -5.26, 1.70 | 0.32 |

a The outcome was the MHI-5 score. The models evaluated data from 12,742, persons and 90,884 observations; on average, participants contributed to 7.1 waves of data collection. The models controlled for age, education, marital status, long-term health conditions, household structure, place of residence, year of data collection, injury (self/family), death (spouse/family), language, and neighbourhood disadvantage. Fixed characteristics such as migrant background were dropped from the main model because that did not change over time.

b Upper and lower confidence intervals at 95% significance.

LR chi2(6) = 34.5, p <0.001

**Table 2.2 Main Effect Model and Interaction Models for Employment and Mental Health by Countries of Origin (SACC) using a Fixed-Effects Regression Model (SACC Level 1- Countries of origin)**

| Variables | Main effect | | | Interaction | | |
| --- | --- | --- | --- | --- | --- | --- |
|  | Coefficient | 95% CI | P value | Coefficient | 95% CI | P-value |
| **Employment Status** |  |  |  |  |  |  |
| Employed | Ref |  |  | Ref |  |  |
| Unemployed | -2.17 | -2.59, -1.75 | <0.001 | -2.13 | -2.59, -1.67 | <0.001 |
| NILF | -1.53 | -1.85, -1.20 | <0.001 | -1.53 | -1.88, -1.17 | <0.001 |
| **Employment status^b^ X countries of origin** |  |  |  |  |  |  |
| Unemployed# English-speaking countries |  |  |  | 0.88 | -0.74, 2.50 | 0.29 |
| Unemployed#North West Europe |  |  |  | 0.05 | -5.99, 6.08 | 0.99 |
| Unemployed#Southern and Eastern Europe |  |  |  | -1.11 | -4.82, 2.60 | 0.56 |
| Unemployed#North Africa and Middle East |  |  |  | -3.54 | -7.40, 0.31 | 0.07 |
| Unemployed#SE Asia |  |  |  | -3.50 | -6.72, -0.29 | 0.03 |
| Unemployed#NE Asia |  |  |  | -2.27 | -6.28, 1.74 | 0.27 |
| Unemployed#SC Asia |  |  |  | -1.95 | -5.54, 1.63 | 0.29 |
| Unemployed#Americas excluding North America |  |  |  | 2.91 | -2.50, 8.32 | 0.29 |
| Unemployed#SSAfrica excluding South Africa |  |  |  | 3.93 | -2.18, 10.03 | 0.21 |
| Unemployed#Oceania |  |  |  | 2.99 | -0.95, 6.94 | 0.14 |
| NILF# English-speaking countries |  |  |  | 0.57 | -0.60, 1.74 | 0.34 |
| NILF#North West Europe |  |  |  | -0.30 | -4.10, 3.50 | 0.88 |
| Variables | Main effect | | | Interaction | | |
|  | Coefficient | 95% CI | P value | Coefficient | 95% CI | P-value |
|  |  |  |  |  |  |  |
| NILF#Southern and Eastern Europe |  |  |  | -3.19 | -5.66, -0.71 | 0.01 |
| NILF# North Africa and Middle East |  |  |  | -0.81 | -4.27, 2.65 | 0.65 |
| NILF#SE Asia |  |  |  | 1.27 | -1.50, 4.04 | 0.37 |
| NILF#NE Asia |  |  |  | 0.86 | -1.73, 3.44 | 0.52 |
| NILF#SC Asia |  |  |  | -0.16 | -3.24, 2.91 | 0.92 |
| NILF#Americas excluding North America |  |  |  | -0.80 | -4.54, 2.95 | 0.68 |
| NILF#SSAAfrica excluding South Africa |  |  |  | -0.78 | -5.90, 4.34 | 0.77 |
| NILF#Oceania |  |  |  | -0.58 | -4.21, 3.05 | 0.75 |

a The outcome was the MHI-5 score. The models evaluated data from **13,031** persons and **94,173** observations; on average, participants contributed to 7.2 waves of data collection. The models controlled for age, education, marital status, long-term health conditions, household structure, place of residence, year of data collection, injury (self/family), death (spouse/family), and neighbourhood disadvantage. Fixed characteristics such as migrant background were dropped from the main model because that did not change over time.

b Upper and lower confidence intervals at 95% significance.

c Referent for the interaction: employed and Australian-born

chi2(6) = 32.5, p-value = 0.04
