# Supplementary material for: Employment-related mental health outcomes among Australian migrants: A 19-year longitudinal study
Source: Aust N Z J Psychiatry. 2023 May 21;57(11):1475–85. doi: 10.1177/00048674231174809 (PMC10619185; doi:10.1177/00048674231174809)
Supplement: sj-docx-3-anp-10.1177_00048674231174809 – Supplemental material for Employment-related mental health outcomes among Australian migrants: A 19-year longitudinal study [file sj-docx-3-anp-10.1177_00048674231174809.docx]

**Supplementary file 3 : Reason for NILF in last four weeks (Top 5)**

|  | Australia | English speaking countries | European countries, excluding ESC | Asia | Middle East and Africa | Oceania and Americas, excluding ESC |
| --- | --- | --- | --- | --- | --- | --- |
| 1. Studying/returning to studies | 37.0 | 18.7 | 14.3 | 42.4 | 24.6 | 36.6 |
| 2. Own illness, injury or disability | 32.2 | 46.1 | 32.1 | 18.8 | 36.9 | 45.1 |
| 3. Does not need to work | 4.4 | 4.9 | 4.8 | 3.5 | 1.5 | 0.0 |
| 4. Caring for a family member | 4.3 | 6.3 | 14.3 | 3.5 | 10.8 | 7.0 |
| 5. Caring for children | 2.8 | 2.1 | 6.0 | 4.7 | 3.1 | 1.4 |
| 6. Other reasons | 19.2 | 21.8 | 28.6 | 27.1 | 23.1 | 9.9 |
